# Granulomatous Vasculitis: An Unusual Manifestation of Metastatic Crohn’s Disease

**DOI:** 10.1111/ajd.70166

**Published:** 2026-06-26

**Authors:** Ethan Wei Min Lee, Helen Yiling Sun, Christopher W. Toon, Lachlan Andrew Byth

**Affiliations:** ^1^ Royal North Shore Hospital Sydney Australia; ^2^ University of New South Wales Sydney Australia

Metastatic Crohn’s disease (MCD) is a rare cutaneous manifestation of Crohn’s disease (CD) characterised by the presence of non‐caseating granulomas in skin lesions that are non‐contiguous with the gastrointestinal tract. MCD has a female predominance and more commonly affects the anogenital region, presenting as fissures and ulcers in this area. On non‐genital skin, MCD usually presents as ulcerating nodules or violaceous plaques. We report the case of MCD that arose during a switch in biologic therapy for longstanding CD.

A 50‐year‐old female with a 26‐year history of ileocolic and perianal CD presented with a 2‐month history of intermittently tender violaceous nodules on her bilateral lower limbs and left upper arm. Two of the largest nodules had recently become superficially infected and were managed with oral antibiotics with her general practitioner. The skin lesions emerged simultaneous with worsening CD symptoms, 1 month into a switch in biologic therapy from adalimumab to ustekinumab. She had concomitant palmoplantar pustulosis and scalp psoriasis thought to be induced by adalimumab, managed with topical steroids.

Physical examination revealed a single nodule on the left upper arm without underlying erythema, and multiple violaceous crusted nodules on the bilateral lower limbs (Figure 1). The differential diagnosis included mycobacterial and deep fungal infection, erythema induratum, MCD, sarcoidosis and lymphoma.

**FIGURE 1:**
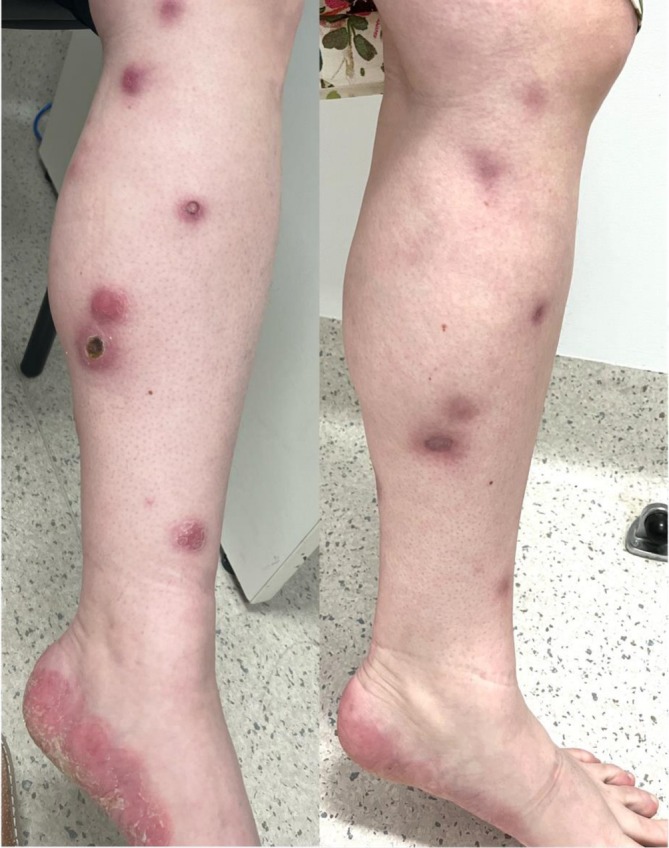
Multiple violaceous nodules at the left medial calf resolving to post‐inflammatory hyperpigmentation.

Punch biopsy of one of the skin nodules revealed superficial and deep dermal perivascular lymphocytic inflammation with striking aggregates of epithelioid histiocytes tightly encasing capillaries and arterioles, which show fibrinoid necrosis (Figure 2). Fat infarction was also noted. No acid‐fast bacilli were seen on Ziehl‐Neelsen and Fite stains. Microbiological cultures for fungi and bacteria were negative. The histopathological findings were consistent with granulomatous vasculitis, a rare and infrequently reported manifestation of MCD [1, 2]. There was no clinical suspicion of alternative causes of vasculitis; thus, no vasculitis work‐up was performed.

**FIGURE 2:**
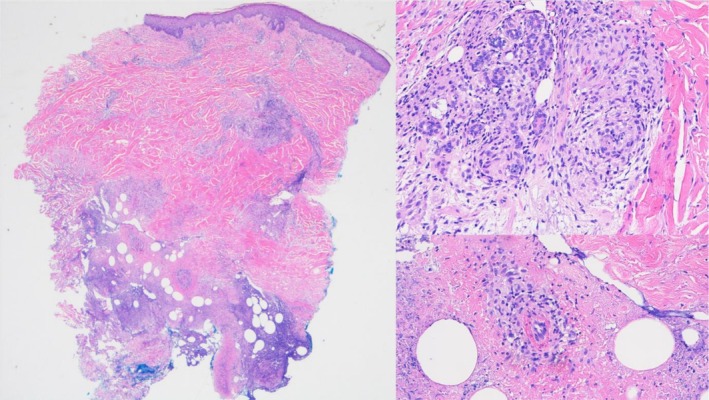
Punch biopsy showed a nodule with superficial and deep dermal perivascular lymphocytic inflammation with aggregates of epithelioid histiocytes tightly encasing capillaries and arterioles, which show fibrinoid necrosis.

The patient was commenced on oral prednisolone 25 mg daily, with clinical improvement of both symptoms and the appearance of lesions noted at 3‐week follow‐up. Complete resolution to post‐inflammatory hyperpigmentation was achieved at 3‐months from initial presentation despite tapering of prednisolone (Figure 1).

Granulomatous vasculitis is an uncommon histological reaction pattern reported in association with ANCA‐associated vasculitis (granulomatosis with polyangiitis and eosinophilic granulomatosis with polyangiitis), large‐vessel vasculitis (giant cell arteritis and Takayasu arteritis), lymphoma (lymphomatoid granulomatosis, angioimmunoblastic T‐cell lymphoma and extranodal NK/T‐cell lymphoma, nasal type), autoimmune disease (MCD, sarcoidosis and rheumatoid arthritis) and infection (tuberculosis, syphilis and post‐herpes zoster) [3]. It is not explicitly recognised as a subtype of vasculitis in the 2012 Revised Chapel Hill Consensus Conference Nomenclature of Vasculitides, but is included in the 2018 Dermatologic Addendum as a pattern of injury with multiple aetiologies [3]. The diagnosis requires direct granulomatous involvement of vessel walls, often with fibrinoid necrosis [4].

It is proposed that the primary disease process in MCD is immune complex‐mediated vasculitis triggering perivascular granuloma formation, known as granulomatous perivasculitis [4]. It is unclear why switching biologic agents appeared to trigger onset of MCD in this case, as previous cases have reportedly responded to ustekinumab [5]. Subtherapeutic drug levels are one possibility, and therapeutic drug monitoring is increasingly used to optimise treatment in CD.

## Conflicts of Interest

The authors declare no conflicts of interest.

## Data Availability

Data sharing not applicable to this article as no datasets were generated or analysed during the current study.
